# Exogenous gibberellin suppressed taproot secondary thickening by inhibiting the formation and maintenance of vascular cambium in radish (*Raphanus sativus* L.)

**DOI:** 10.3389/fpls.2024.1395999

**Published:** 2024-09-12

**Authors:** Ge Meng, Mingli Yong, Ziyue Zhang, Yuqing Zhang, Yahui Wang, Aisheng Xiong, Xiaojun Su

**Affiliations:** ^1^ Jiangsu Key Laboratory for Horticultural Crop Genetic Improvement, Institute of Vegetable Crops, Jiangsu Academy of Agricultural Sciences, Nanjing, China; ^2^ State Key Laboratory of Crop Genetics and Germplasm Enhancement, Ministry of Agriculture and Rural Affairs Key Laboratory of Biology and Germplasm Enhancement of Horticultural Crops in East China, College of Horticulture, Nanjing Agricultural University, Nanjing, China; ^3^ College of Life Sciences, Jiangsu University, Zhenjiang, China; ^4^ College of Horticulture, Anhui Agricultural University, Hefei, China

**Keywords:** *Raphanus sativus*, gibberellin, taproot thickening, transcriptomic analyses, vascular cambium

## Abstract

**Introduction:**

The thickening of radish taproots is primarily determined by secondary growth driven by the vascular cambium and is a highly intricate process regulated by plant hormones, transcription factors, and many metabolic pathways. Gibberellin (GA), a plant hormone associated with cell elongation, is essential in secondary growth. However, the mechanism through which exogenous GA3 regulates secondary taproot growth in radishes remains unclear.

**Methods:**

Integrated morphological, anatomical, hormonal, and transcriptomic analyses of taproots in radishes treated with GA3 and its biosynthesis inhibitor paclobutrazol (PBZ) were performed to explore their effects on taproot secondary growth and key regulatory pathways.

**Results:**

GA3 significantly hindered taproot thickening by inhibiting the formation and maintenance of the vascular cambium, and PBZ promoted root development by increasing root length rather than root diameter. Transcriptome analysis revealed 2,014, 948, and 1,831 differentially expressed genes identified from the control vs. GA3, control vs. PBZ, and GA3 vs. PBZ comparisons, respectively. Kyoto Encyclopedia of Genes and Genome pathway enrichment analysis revealed that differentially expressed genes were primarily involved in the biosyntheses of secondary metabolites and metabolic pathways. GA3 significantly increased the levels of endogenous indole-acetic acid and the expression of auxin synthesis and signal transduction genes.

**Discussion:**

Exogenous GA3 significantly inhibited the expression of genes involved in the maintenance and differentiation of vascular cambium, including WOX14, ER/ERL1, and XCP2. Exogenous GA3 affects root thickening in radishes primarily by regulating hormone signal transduction pathways, vascular cambium activity, and substance and energy metabolisms. Our findings provide insights into the mechanisms underlying taproot thickening in radishes and provide a valuable gene database for future studies.

## 1 Introduction

Radish (*Raphanus sativus* L.) belongs to the family Brassicaceae and is an economically essential root crop in East Asia because of its edible fleshy taproot rich in water, carbohydrates, proteins, and secondary metabolites (Lu et al., 2008; Jang et al., 2015). Radish storage roots are formed by the thickening of the hypocotyl and upper roots (Zaki et al., 2012). Root thickening, also known as secondary or radial growth, is the most critical stage for final productivity and quality formation (Jang et al., 2015). Therefore, studies have focused on understanding the physiological mechanisms underlying secondary growth. Taproot thickening is primarily owing to the activity of the vascular cambium and the differentiation of the secondary xylem and phloem (Tonn and Greb, 2017), determined primarily by endogenous and environmental factors, including temperature, light, nutrient, water supply, and hormones (Matveeva et al., 2004; Choi et al., 2011; Yu et al., 2015).

Phytohormones are critical for regulating different aspects of secondary growth (Ragni and Greb, 2018). Gibberellins (GAs) are reportedly crucial in secondary growth (Fischer et al., 2019). In Populus, GC/MS-SRM analysis revealed that bioactive GA_1_ and GA_4_ are predominantly in the zone of xylem cell expansion and are crucial in regulating the early stages of xylem differentiation (Israelsson et al., 2005). Transgenic approaches have shown that the overexpression of the GA-related biosynthesis gene, GA20ox, and signaling genes, GID1 and MYB221, facilitates secondary growth and biomass in trees (Mauriat and Moritz, 2009; Cho et al., 2019). Furthermore, GA_3_ application induced the expression of genes related to xylem development in *Eucalyptus grandis* (Liu et al., 2018) and *Betula platyphylla* (Guo et al., 2015). These positive GA effects on secondary growth are not restricted to woody species, as described for *Arabidopsis* (Ragni et al., 2011) and rice (Qin et al., 2022). A study on mutant and transgenic plants in *Arabidopsis* has shown that GA and its signaling pathway are essential and sufficient to enhance secondary growth directly (Ragni et al., 2011). Further advances in understanding the role of GA in regulating vascular secondary activity have revealed that GA signaling promotes cellulose synthesis by relieving the interaction of the GID1-DELLA-NAC signaling cascade in rice and *Arabidopsis* (Huang et al., 2015).

The effects of GAs on regulating root formation have been recently investigated in several tuber/root crops (Matveeva et al., 2004; Jung and McCouch, 2013), which depended on the studied species. Exogenous GA_4/7_ stimulated stolon elongation and inhibited tuber expansion in potatoes (Xu et al., 1998), whereas exogenous GA_3_ significantly increased tuber weight and yield in yam (Gong et al., 2015). Exogenous application of GA_3_ to sweet potato shoots increases root GA levels, promotes root lignification, and prevents a shift from roots to storage root organs, causing reduced storage root number and diameter (Singh et al., 2019). Similar to potatoes and sweet potatoes, GA negatively affects taproot development because GA_3_ induces root elongation and produces thinner roots in carrots and turnips (Wang et al., 2015; Liu et al., 2021). These studies show that GAs regulate tuber/root growth differently. The direct study of the distinct roles of GAs in root development in radishes is lacking, and the underlying physiological and molecular regulatory mechanisms associated with GA-regulated taproot thickening remain unclear. In this study, exogenous GA_3_ and its biosynthesis inhibitor, paclobutrazol (PBZ), were applied to radish seedlings, and their phenotypic data, anatomical change, endogenous GA_3_ and IAA content and gene expression profiles were then comprehensively analyzed to explore the role of GA_3_ in the radish root thickening. Our study firstly revealed that GA_3_ inhibited taproot secondary growth by affecting the hormone synthesis and signal transduction pathways and inhibiting the occurrence and maintenance of vascular cambium. Our study provided valuable information for further molecular mechanism analysis behind GA-dependent taproot thickening in radish and offered a biomass engineering direction for controlling yields.

## 2 Results

### 2.1 Exogenous GA_3_ inhibited taproot thickening in radish

**Figure 1:**
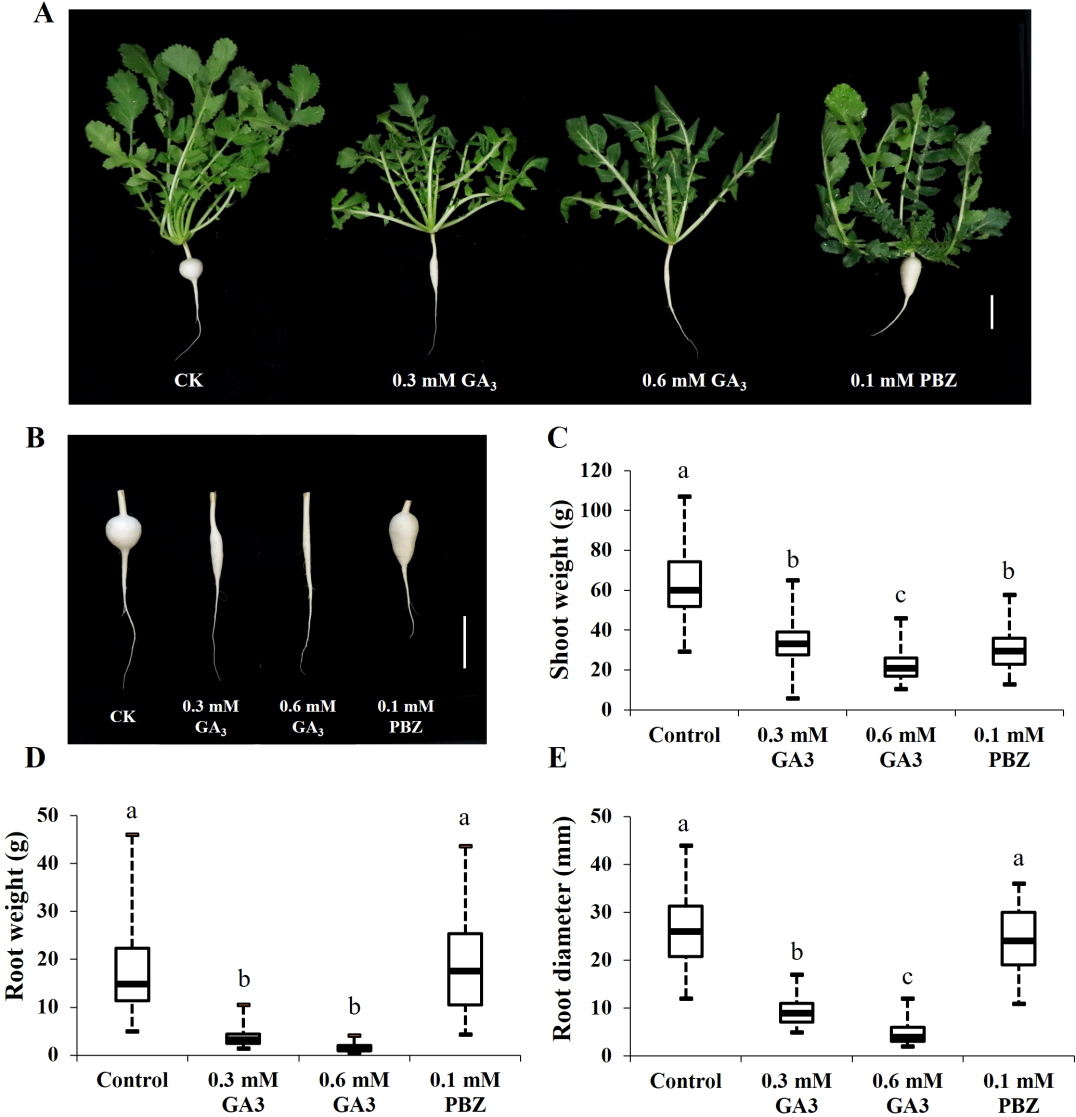
Effects of GA_3_ and PBZ on radish seedlings. Phenotypes changes of 21-day-old radish seedlings **(A)** and taproots **(B)**after two weeks under different treatments. Statistical analysis of the effects of GA_3_ and PBZ on radish shoot weight **(C)**, root weight **(D)**, and root diameter **(E)**. The data are presented as the means ± SEs (n = 20), error bars indicate error standard. Different letters indicate significant differences at p < 0.05. CK, control; GA_3_, exogenous GA_3_ treatment; PBZ, exogenous PBZ treatment. Scale bar = 5 cm.

The cortex split of radish roots was achieved approximately 21 days after sowing, after which taproot thickening rapidly expanded (Wang et al., 2013; Yu et al., 2016). Based on this, to explore the effects of GA on taproot thickening, GA_3_ (0.3, 0.6 mM) and 0.1 mM PBZ, a GA biosynthesis inhibitor, were prepared and sprayed onto the leaf of 3-week-old radish seedlings every 2 days, and water-treated seedlings were used as a reference for normal growth (CK). The root and shoot fresh weights and root circumference of the seedlings under different treatments were measured separately after 2 weeks. Shoot weight, root weight, and root diameter of radish seedlings treated with GA_3_ were significantly lower than those of the controls, and GA_3_ inhibited radish seedling growth in a dose-dependent manner (**Figure 1**). In addition, PBZ significantly increased root weight by increasing root length; however, it had no significant effects on root diameter (**Figure 1**). These results indicate that exogenous GA_3_ strongly inhibited taproot thickening, and exogenous PBZ changed the root shape in the radish.

### 2.2 Exogenous GA_3_ inhibited the formation and maintenance of vascular cambium

**Figure 2:**
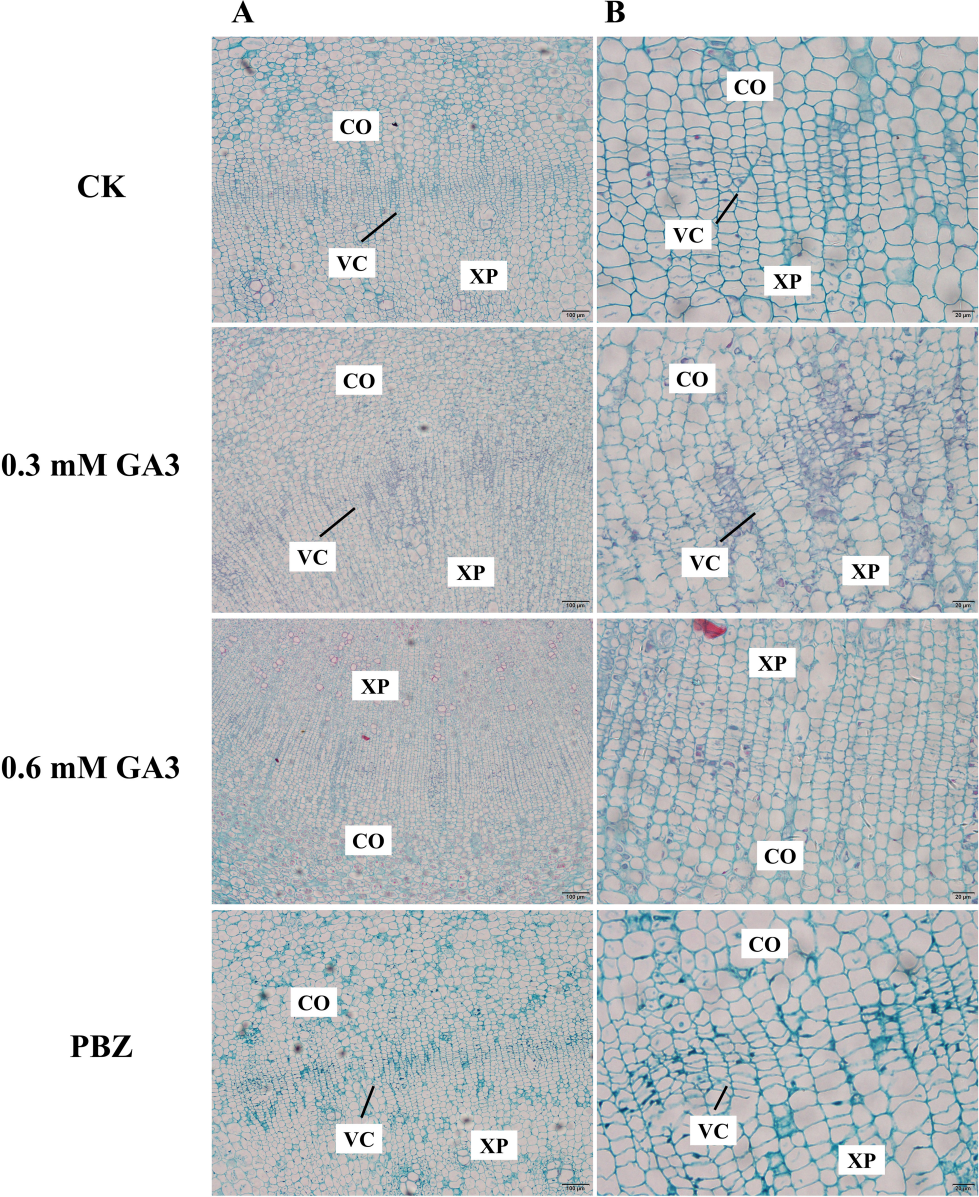
Anatomical structure analysis of radish taproots treated with GA_3_ and PBZ. Cross-section images of roots treated with CK, 0.3 mM GA_3_, 0.6 mM GA_3_, 0.1 mM PBZ. CO: root cortex, XP: xylem parenchyma, VC: root vascular cambium, VE: vessel. Scale bars = 100 µm **(A)** and 20 µm **(B)**.

Previous studies have shown that cambial cell division is closely correlated with radial root growth in radish (Jang et al., 2015). In this case, we expected that the phenotypic differences in radish taproots with different treatments were caused by differences in cell division activities in the cambium. To test our hypothesis, we observed the anatomical structure of taproots under different treatments for 2 weeks using histological sections. Cambial zone is the opaque regions that are composed of layers of thin cells that are stacked in varying numbers. Taproots treated with water and PBZ had a well-established vascular cambium, and their vascular structures were clear and ordered (**Figure 2**); However, the number of cells inside the cambial layer in the taproots of plants treated with GA_3_ was significantly lower than that in the control plants; There was almost no cambial zones observed in taproots treated with 0.6 mM GA_3_. The results indicate that exogenous GA_3_ inhibited the formation and maintenance of the vascular cambium, causing the inhibition of taproot thickening in radish.

### 2.3 Changes in endogenous GA_3_ and indole-acetic acid contents under GA_3_ and PBZ treatments

**Figure 3:**
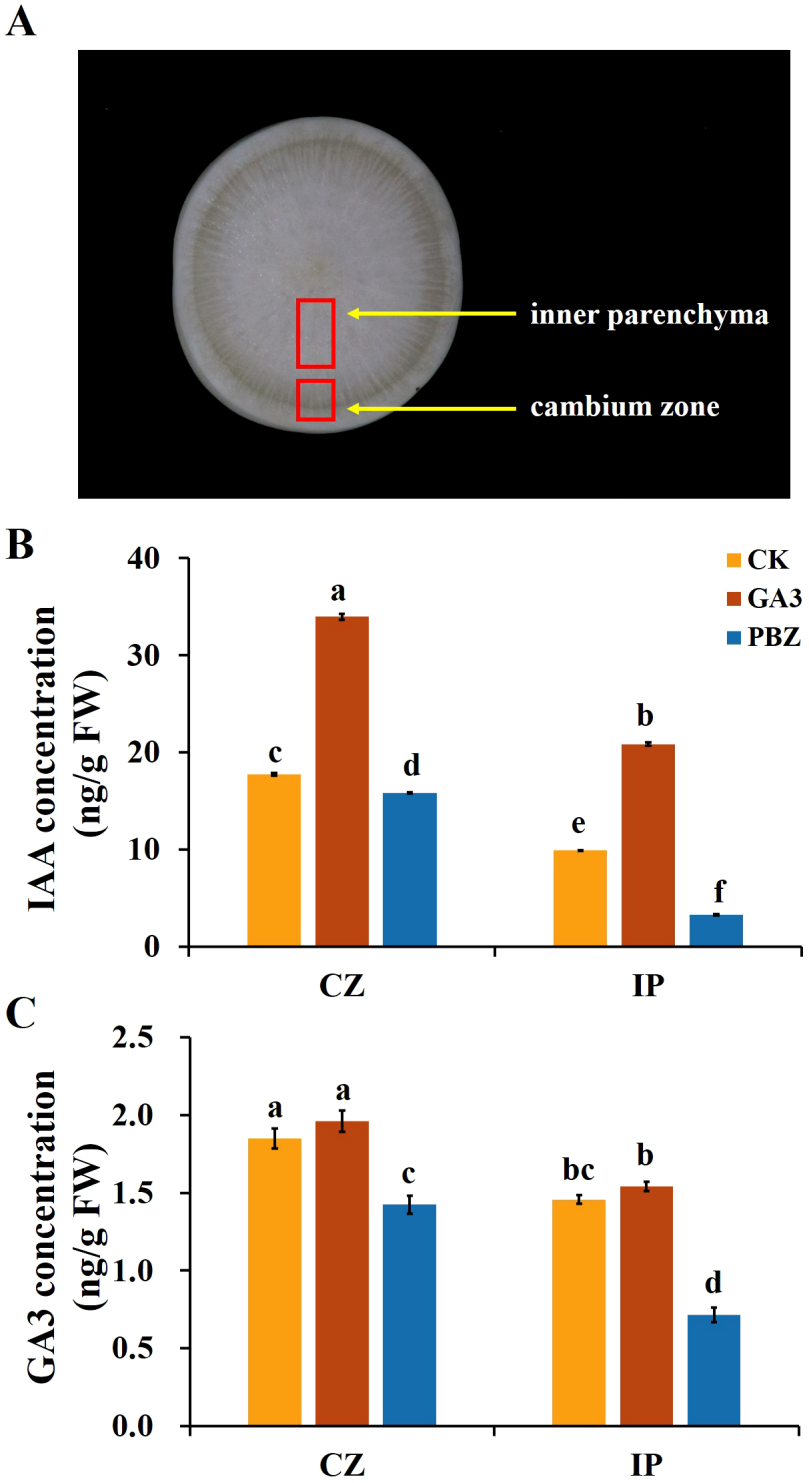
Endogenous indole-3-acetic acid (IAA) and GA_3_ contents of taproots in radish under different treatments. **(A)** Image showing the cambium zone (CZ) and inner parenchyma (IP) regions of taproot used for the analysis of GA_3_ and IAA content. The endogenous IAA **(B)** and GA_3_ **(C)** concentration in the taproot under different treatments. CK, control; GA_3_, exogenous GA_3_ (0.3 mM) treatment; PBZ, exogenous PBZ treatment. The data are presented as the means ± SEs (n = 3).

Plant hormones are the essential internal regulators of secondary growth. The exogenous application of GA_3_ and PBZ significantly affected taproot secondary growth in radish (**Figure 1**), prompting the exploration of the effect of exogenous GA_3_ and PBZ treatments on the endogenous hormone content. Previous studies have shown that auxin and its signaling processes promote cambial activity and vascular patterning (Miyashima et al., 2013). Therefore, in addition to the effects of exogenous GA_3_ (0.3 mM) and PBZ treatments on endogenous GA_3_ content in radish roots, we focused on their effects on endogenous IAA content. Two regions, the cambium zone and inner parenchyma, of roots treated with GA_3_ and PBZ after 2 weeks were collected to extract GA_3_ and IAA for content analysis using high-performance liquid chromatography (HPLC). The IAA and GA_3_ contents in the cambium zone region of the control group were significantly higher than those in the internal parenchyma region (**Figure 3**). Exogenous GA_3_ significantly increased endogenous IAA and GA_3_ contents, particularly IAA, in the cambium zone and internal parenchyma, compared with the control. However, the opposite trend for IAA and GA_3_ contents was observed in the roots treated with PBZ. The IAA and GA_3_ contents of PBZ-treated roots were decreased compared with those of the control in both regions, and the IAA content in the internal parenchyma decreased markedly. These results indicate that the exogenous application of GA_3_ and PBZ changed the endogenous GA_3_ and IAA levels, particularly that of IAA.

### 2.4 Transcriptome analysis

**Figure 4:**
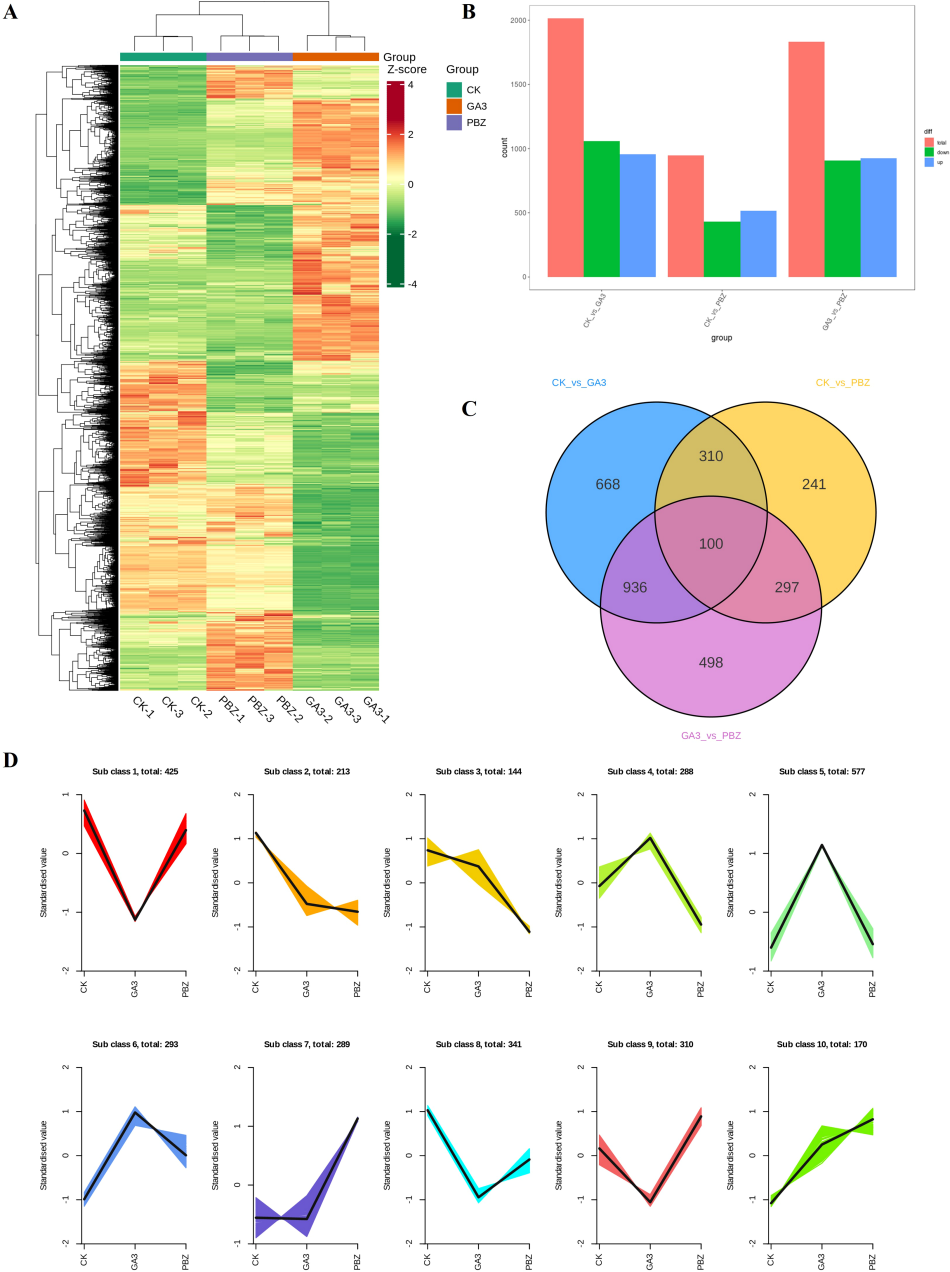
Overview of differentially expressed genes (DEGs) in radish roots under different treatments. **(A)** Hierarchical clustering of DEGs of all samples. The red grids indicate up-regulation of expression, while the green grids indicate downregulation of expression. **(B)** Numbers of upregulated and downregulated genes identified from the different comparisons. **(C)** Venn diagram of commonly and exclusively expressed DEGs in each comparison group. **(D)** K-means clustering of DEGs expression trends, the expression profiles of genes in each subclass are represented by different colors, and the average expression profiles of all genes in each sample are represented by a black line.

Transcriptome analysis was conducted to explore the mechanism by which GA_3_ and PBZ regulate the process of radish root thickening. Based on the phenotypic analysis, taproot samples treated with GA_3_ (0.3 mM), PBZ, and the control groups were used to construct nine libraries for sequencing. In total, 543,731,994 raw reads were produced, 508,962,020 qualified reads were obtained, 430,191,565 reads (approximately 84.5%) were mapped, and 410,223,047 reads (approximately 80.6%) were uniquely mapped to the radish reference genome. Over 96.67% of the clean reads were at the Q20 level, and over 91.29% of the clean reads were at the Q30 level (detailed results are presented in **Supplementary Table S1**). Principal component analysis showed that biological replicates from the same treatment group were highly correlated, and different treatments were separated from each other (**Supplementary Figure S1**). Global comparisons of the gene expression profiles of the taproots under different treatments are shown in a heat map, with good repeatability for each treatment and large transcriptional differences between the groups (**Figure 4A**). GA_3_ and PBZ treatments caused a substantial change in gene expression compared with the CK treatment.

A pairwise differential expression profiling analysis was performed to identify differentially expressed genes (DEGs) under different treatments (**Figure 4B**). Compared with the control, there were 2,014 (954 upregulated DEGs and 1,060 downregulated DEGs) and 948 DEGs (518 upregulated DEGs and 430 downregulated DEGs) existed in GA_3_ and PBZ treatment samples, respectively. Meanwhile, compared with the GA_3_ treatment samples, there were 1,831 DEGs (925 upregulated DEGs and 906 downregulated DEGs) existed in GA_3_ and PBZ treatment samples. The Venn diagram demonstrating DEGs among the three groups revealed that most of the DEGs were uniquely associated with a specific treatment and that there were 100 common DEGs among the three comparison groups (**Figure 4C**). The list of the detected DEGs is presented in **Supplementary Table S2**. All identified 3,050 DEGs were classified into 10 subclasses using the K-means clustering method based on similarities in gene expression profiles (**Figure 4D**). Subclass 2 contained the most DEGs, reaching 577, which were markedly induced by GA_3_. The expression levels of 288 DEGs in Subclass 4 increased under GA_3_ treatment but decreased under PBZ treatment, and the expression trend of DEGs in Subclass 9 demonstrated an opposite pattern to that in Subclass 4.

### 2.5 Gene ontology enrichment analysis

**Figure 5:**
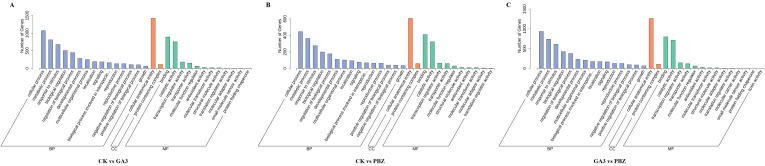
Functional classification of DEGs according to GO analysis. GO analysis of DEGs identified from the comparison of the CK *vs.* GA_3_ group **(A)**, CK *vs.* PBZ group **(B)**, and GA_3_ *vs.* PBZ group **(C)**. The x-axis represents the name of the GO terms. The y-axis represents the total number of annotated DEGs for each GO term.

The DEGs identified from the comparison of the CK *vs.* GA_3_ group were enriched in 41 GO terms, which included 25 biological process (BP), 2 cellular component (CC), and 14 molecular function (MF) terms (**Figure 5A**). We selected the top 20 GO terms with enrichment numbers for further analysis and found that “phosphorelay signal transduction system”, “cellular response to ethylene stimulus”, “ethylene-activated signaling pathway”, and “cellular response to extracellular stimulus” were significantly enriched. Of the DEGs identified from the CK *vs.* PBZ group, 948 DEGs were significantly enriched in 35 GO terms, which included 20 biological process (BP), two cellular component (CC), and 13 molecular function (MF) terms (**Figure 5B**). We selected the top 20 GO terms with enrichment numbers for further analysis and found that “response to insect”, “regulation of proteolysis”, “regulation of hydrolase activity” and “negative regulation of molecular function” were significantly enriched. Of the DEGs identified from the GA_3_ *vs.* PBZ group, 1,831 DEGs were significantly enriched in 40 GO terms, which included 23 biological process (BP), two cellular component (CC), and 15 molecular function (MF) terms (**Figure 5C**). We selected the top 20 GO terms with enrichment numbers for further analysis and found that “response to toxic substance”, “response to hypoxia”, “response to decreased oxygen levels”, and “response to oxygen levels” were significantly enriched. These results indicated that a large number of genes regulated by GA_3_ and PBZ treatments were involved in taproot thickening.

### 2.6 Kyoto Encyclopedia of Genes and Genomes enrichment analysis of DEGs

**Figure 6:**
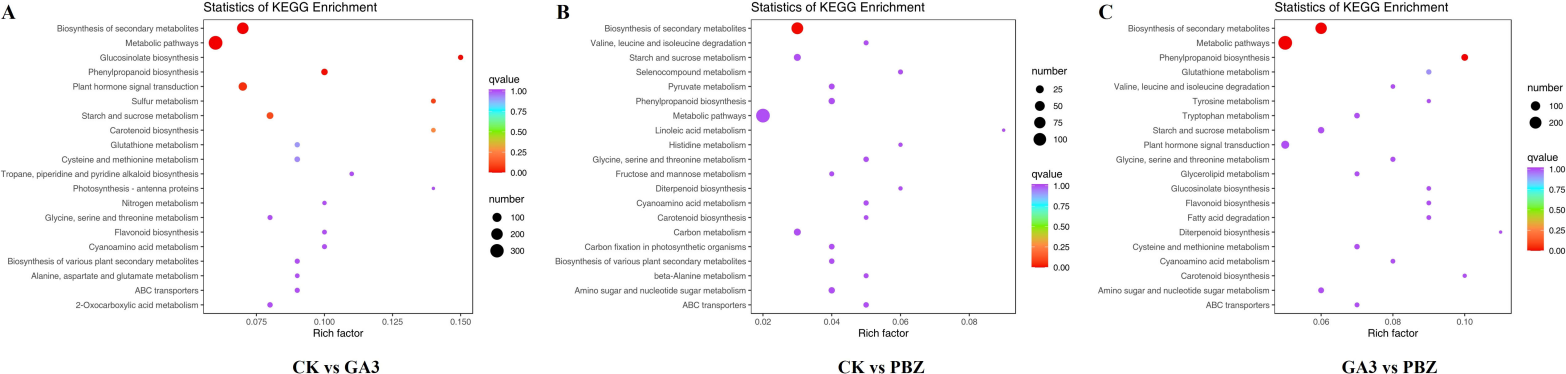
The top 20 of KEGG enrichment pathways of DEGs identified from each comparison group. KEGG pathway enrichment scatter plot of the comparison of the CK *vs.* GA_3_ group **(A)**, CK *vs.* PBZ group **(B)**, and GA_3_ *vs.* PBZ group **(C)**. The y-axis is the pathway, and the x-axis is the percentage of this pathway of the total Rich Factor. The darker the color, the smaller the Q value and the higher the enrichment degree. The size of the dots indicates the number of DEGs in this pathway.

To identify the primary pathways through which GA_3_ and PBZ contribute to root thickening in radish, DEGs were subjected to KEGG pathway enrichment analysis. DEGs were identified to be involved in 126, 100, and 124 metabolic pathways in the control and GA_3_-treated groups (CK *vs.* GA_3_), the control and PBZ-treated groups (CK *vs.* PBZ) and the GA_3_ and PBZ-treated groups (GA_3_ *vs.* PBZ), respectively. We selected the top 20 of each comparison group for further analysis, comprising 35 pathways (**Figure 6**). The pathways significantly enriched in the three comparison groups were biosynthesis of secondary metabolites (ko01110), metabolic pathways (ko01100), glycine, serine and threonine metabolism (ko00260), cyanoamino acid metabolism (ko00460), starch and sucrose metabolism (ko00500), carotenoid biosynthesis (ko00906), phenylpropanoid biosynthesis (ko00940), and ABC transporters (ko02010). Moreover, plant hormone signal transduction pathway (ko04075), flavonoid biosynthesis (ko00941), glucosinolate biosynthesis (ko00966), glutathione metabolism (ko00480), and cysteine and methionine metabolisms (ko00270) were specifically enriched in the CK *vs.* GA_3_ and GA_3_ vs PBZ groups; amino sugar and nucleotide sugar metabolisms (ko00520), valine, leucine, and isoleucine degradations (ko00280), and diterpenoid biosynthesis (ko00904) were specifically enriched in the CK *vs.* PBZ and GA_3_ *vs.* PBZ groups; biosyntheses of various plant secondary metabolites (ko00999) were specifically enriched in the CK *vs.* GA_3_ and GA_3_ *vs.* PBZ groups. These results suggest that GA_3_ and PBZ affect root thickening in radishes primarily by regulating hormone signal transduction pathways and substance and energy metabolisms.

### 2.7 DEGs related to hormone biosynthesis and signal transduction pathways

**Figure 7:**
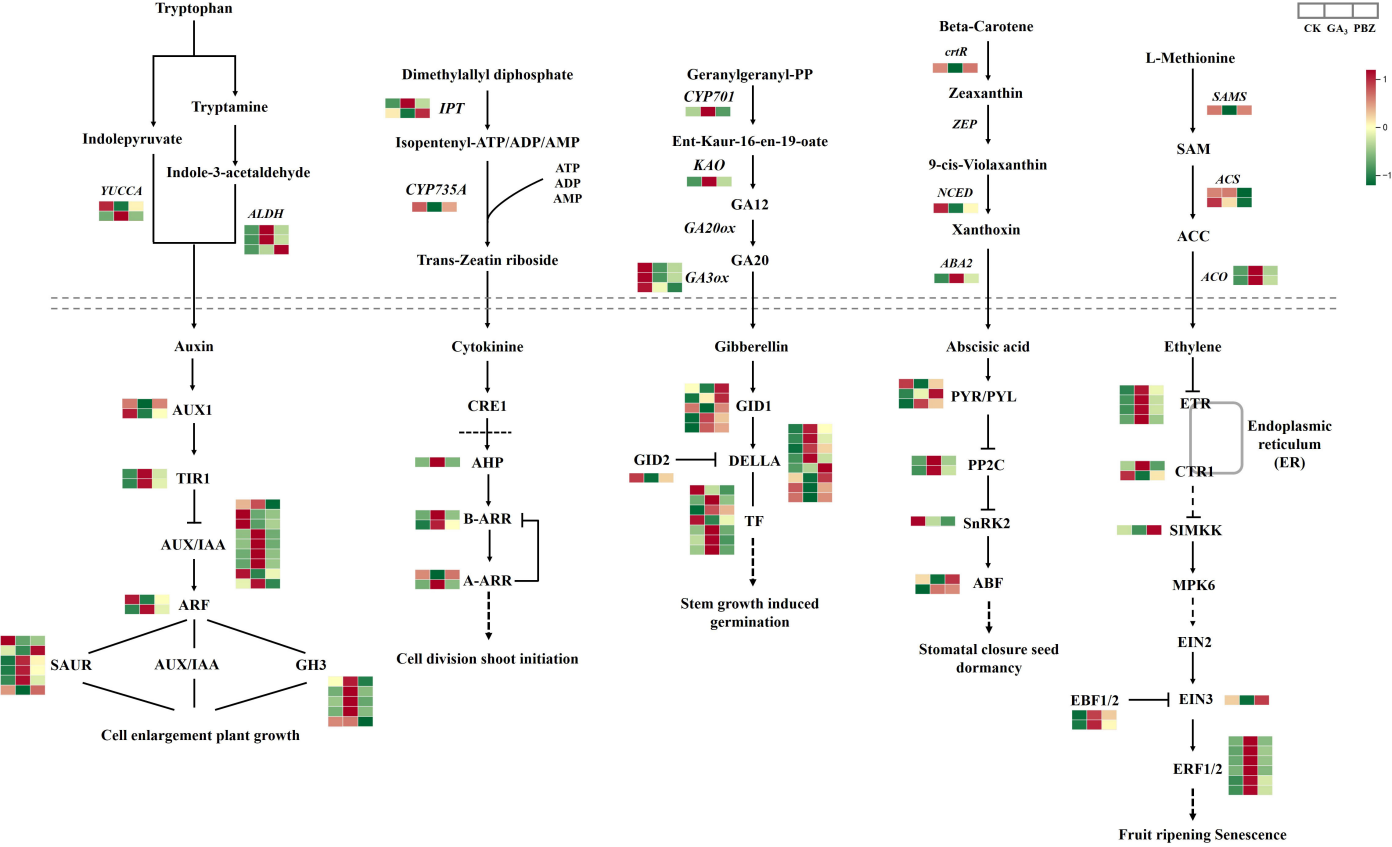
DEGs involved in the synthetic metabolic pathway of auxin, cytokinin, gibberellin, abscisic acid, and ethylene under different treatments. Above the dotted line is the synthetic pathway, and below the dotted line is the metabolic pathway. Different colors show the value of the fold change, and the red indicates upregulated expression, and green indicates downregulated expression in the heatmap. The order of sample was shown on the top right. CK, control; GA_3_, exogenous GA_3_ (0.3 mM) treatment; PBZ, exogenous PBZ treatment.

To examine the effect of GA_3_ and PBZ treatments on the hormone biosynthesis and signal transduction pathway in radish, we examined the expression of DEGs involved in auxin, cytokinin, gibberellin, abscisic acid, and ethylene biosyntheses and signal transduction pathways. We identified 97 DEGs (**Figure 7**; **Supplementary Table S3**). Auxin and its signaling processes reportedly promote cambial activity and vascular patterning (Miyashima et al., 2013). Abundant DEGs were involved in auxin synthesis and signal transduction pathways. The expression patterns of most genes involved in the auxin synthesis pathway were induced by exogenous GA_3_, including aldehyde dehydrogenase NAD+ (ALDH) and indole-3-pyruvate monooxygenase (YUCCA), causing marked accumulation of IAA, which is consistent with the examination of the IAA content mentioned above (**Figure 3B**). The expression profiles of most genes involved in the auxin signal transduction pathway were similar to those of auxin synthetic genes; the highest expression levels of genes, including transport inhibitor response (TIR1), auxin-responsive protein (AUX/IAA), auxin-responsive gene (GH3), and auxin-induced protein (SAUR), were identified under GA_3_ treatment. These results indicate that auxin synthesis and signal transduction pathways are induced by GA_3_ and that the regulatory pathways initiated by auxin might enhance the negative effect of GA_3_ on root thickening in radish.

Similarly, cytokinins (CKs) are positive signals of cambial activity (Matsumoto-Kitano et al., 2008). In the CKs synthesis pathway, two genes encoding adenylate dimethylallyltransferase (IPT) showed different expression patterns under GA_3_ and PBZ treatments. Cytokinin transhydroxylase (CYP735A) was suppressed through GA_3_ and PBZ treatments. In addition, we examined the HISTIDINE-CONTAINING PHOSPHOTRANSMITTER gene (AHP), TYPE-A ARABIDOPSIS RESPONSE REGULATOR (A-ARR), and TYPE-B ARABIDOPSIS RESPONSE REGULATOR (B-ARR) to detect the cytokinin signaling pathways in response to GA_3_ and PBZ treatments. These genes were induced; however, only one member of the A-ARR family was suppressed by GA_3_, and PBZ application had no significant effect on their expression.

The expression profiles of genes, including CYP701 and KAO involved in the GA synthesis pathway, were significantly upregulated after GA_3_ treatment, promoting the high synthesis of GA12. The expression levels of genes encoding GA3ox3, a significant enzyme in the last step of gibberellin biosynthesis, were significantly down-regulated after treatment with GA_3_ and PBZ. Despite this, GA_3_ content increased after its application. Therefore, in the gibberellin signaling pathway, the expression of most genes encoding the GA receptor GID1, DELLA protein, and phytochrome-interacting factor (TF) was induced by GA_3_ and PBZ. Similarly, we evaluated the expression level of GID2, a gene responsible for GA inactivation, and found that GID2 expression was suppressed by GA_3_ and PBZ (**Figure 7**).

Genes involved in ABA biosynthesis were differentially expressed in our data, including beta-carotene 3-hydroxylase (crtR), Nine-cis-epoxycarotenoid dioxygenase (NCED), and ABA DEFICIENT 2 (ABA2). The crtR and NCED were repressed by GA_3_ treatment. In contrast, ABA2 expression was induced by 2.10- and 1.39-folds after GA_3_ and PBZ treatments, respectively, promoting the synthesis of abscisic aldehyde and establishing sufficient precursor substances for the accumulation of ABA. Most DEGs involved in the ABA signal transduction pathway, including the ABA receptors PYR/PYL and protein phosphatase 2C, were induced by GA_3_ and PBZ treatments. A member of the PYR/PYL was significantly repressed after GA_3_ and PBZ treatment, particularly with GA, and serine/threonine-protein kinase (SnRK2) was repressed by GA_3_ and PBZ treatments. The ABA-responsive element-binding factor included two members that showed different expression patterns under GA_3_ and PBZ treatments. One member was repressed by GA_3_ but not affected by PBZ, while the other was induced by GA_3_ and PBZ.

S-adenosyl-L-methionine (SAM) synthetase, 1-aminocyclopropane-1-carboxylic acid (ACC) synthases, and ACC oxidases are the key enzymes involved in ET biosynthesis. Of these, the expressions of SAMS and two ACC synthase genes were inhibited using GA_3_ and PBZ, respectively, and two genes encoding ACC oxidase were induced using GA_3_ and PBZ, particularly GA_3_, causing significant accumulation of ETH. In the ETH signal transduction pathway, the expression levels of various ETH receptors, Ethylene response 1, Ethylene-response factors, and Ethylene-Insensitive 3 binding F-box protein1/2 were higher under GA_3_ and PBZ treatments. Two genes encoding Constitutive triple response 1 displaced different expression patterns under GA_3_ and PBZ treatments. The expressions of mitogen-activated protein kinase kinase (SIMKK) and Ethylene-Insensitive 3 were inhibited by GA_3_ and induced by PBZ. Plant hormone metabolism, particularly auxin synthesis and signal transduction pathways, are involved in taproot thickening regulated by GA_3_ in radish.

### 2.8 DEGs are related to the formation and maintenance of vascular cambium

**Figure 8:**
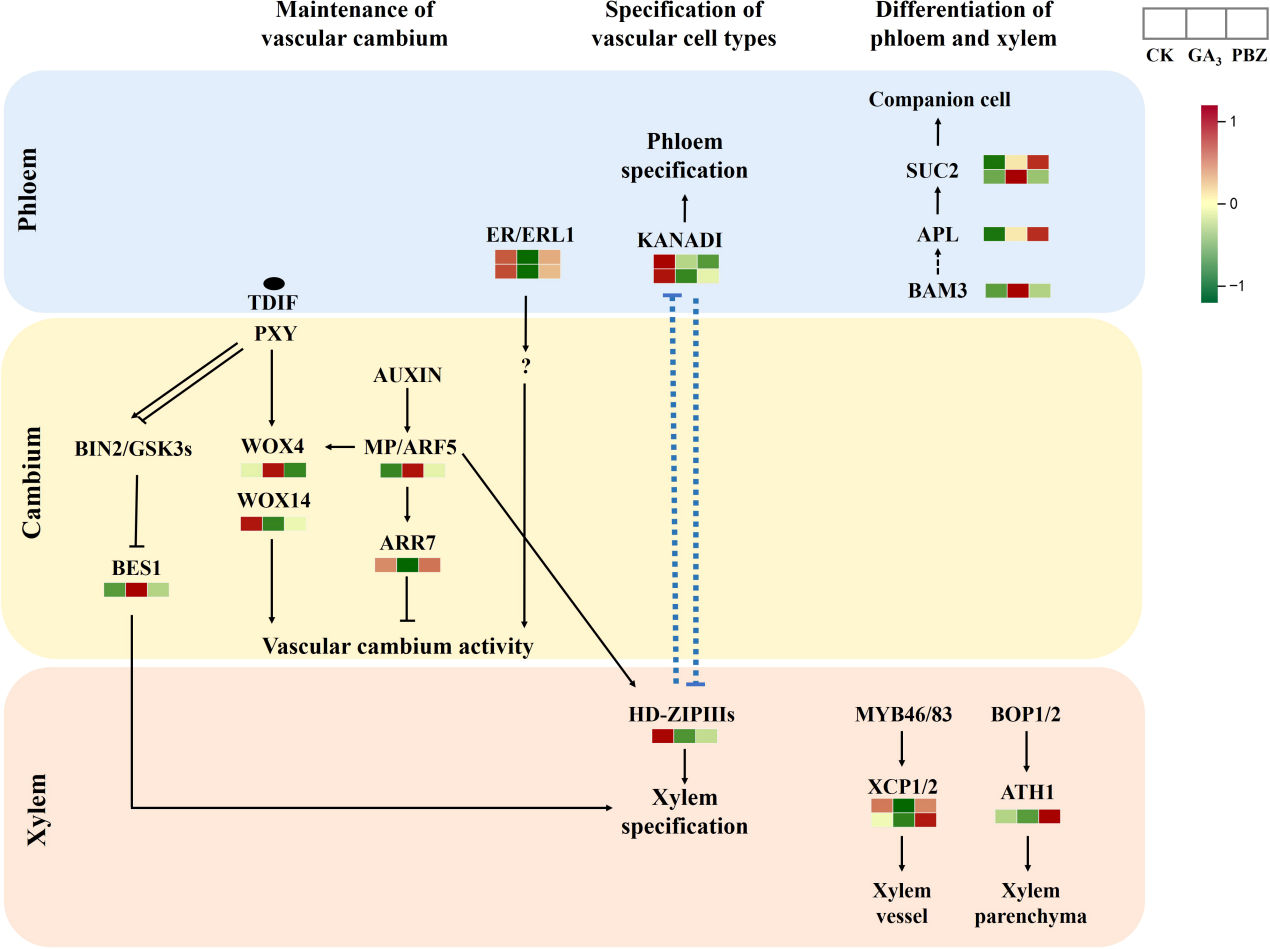
Expression patterns of radish genes that are homologous to *Arabidopsis* genes known for vascular cambium maintenance and differentiation. Different colors show the value of the fold change, and the red indicates upregulated expression, and green indicates downregulated expression in the heatmap. The order of sample was shown on the top right.

The current understanding of the molecular network regulating secondary growth results primarily from studies on *Arabidopsis*. Therefore, we examined the expression patterns of the putative orthologs of key cambium regulators and hormone signaling components that control vascular tissue development in *Arabidopsis* (**Figure 8**; **Supplementary Table S4**). The Phloem Intercalated with Xylem/Tracheary Element Differentiation Inhibitory Factor Receptor (PXY/TDIF) signaling network regulates cambium proliferation and vascular patterning (Hirakawa et al., 2010; Etchells et al., 2013). Downstream of PXY/TDIF signaling, WUSCHEL-related homeobox4 (WOX4) and WOX14 redundantly promote cambial activity (Etchells et al., 2013). The two WOX transcription factors exhibited different expression patterns under GA_3_ and PBZ treatments. Xylem cell differentiation is also regulated by the PXY/TDIF module; however, through a different pathway not involving WOX4. The GLYCOGEN SYNTHASE KINASE 3 proteins interact with PXY and repress the transcription factor BRI1-EMS SUPPRESSOR 1 (BES1), consequently inhibiting xylem cell differentiation (Kondo et al., 2014). The genes encoding GLYCOGEN SYNTHASE KINASE 3 protein showed high expression levels after treatment with GA_3_ and PBZ. In addition to the TDIF-PXY module, the signaling pathway initiated by the EPFL4/6-ER module regulates cambium activity. ERECTA (ER)-LIKE1 (ERL1) and ERL2 function redundantly with ER and comprise the ER family, which positively regulates cambium activity (Shpak et al., 2004). In this study, ERL1 expression was inhibited by GA_3_ and PBZ treatments. These expression dynamics collectively indicate that exogenous GA_3_ significantly affects the activity of the vascular cambium and that DEGs are potentially involved in radish taproot thickening regulated by GA_3_.

### 2.9 Gene expression analysis of RNA-seq data

**Figure 9:**
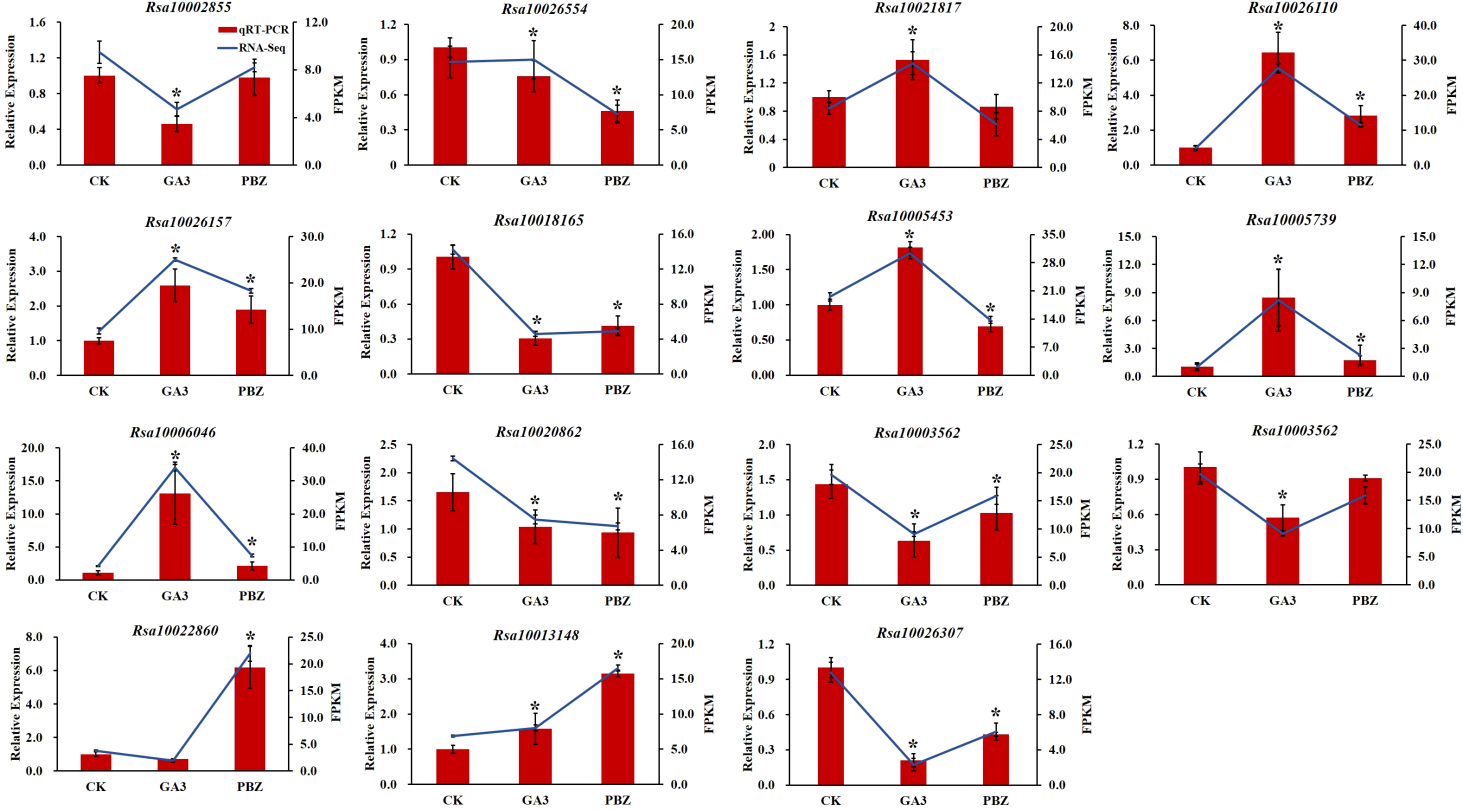
Validation of the transcriptomic data with RT-qPCR. The trend of DEGs expression in transcriptome (blue lines) and its RT-qPCR validation (red bars). FPKM (fragments per kilobase of transcript per million fragments mapped) values were used to represent the relative expression of genes in transcriptome. Error bars show the standard error between three biological replicates performed (n=3). Asterisks (*) denote significant differences compared to CK based on independent-samples *t*-test and significance was determined at P<0.05.

To validate the reliability of the transcriptomic data, 15 DEGs were randomly selected for reverse transcription quantitative real-time polymerase chain reaction (RT-qPCR) analysis. The expression trend found for the DEGs in the RNA-Seq analysis was basically consistent with that obtained by RT-qPCR (**Figure 9**). Therefore, RT-qPCR analysis confirmed the validity of the transcriptome data, indicating that they were reliable and accurate.

## 3 Discussion

### 3.1 GA_3_ retarded taproot thickening by inhibiting the formation of vascular cambium

Radish, a highly marketable winter vegetable, is characterized by an enlarged fleshy taproot with huge variations in shapes and sizes (Zaki et al., 2012). The secondary root thickening stage, primarily involving root expansion, is the most critical period for the final productivity and quality formation in radishes (Yu et al., 2015). Therefore, clarifying the molecular genetic mechanisms underlying taproot thickening and isolating the key genes controlling their taproot thickening are crucial for better or sustainable yields. Several studies have been conducted on the morphological, physiological, and anatomical characterization of taproot thickening in radishes (Zaki et al., 2012; Jang et al., 2015). In addition, integrative transcriptomic and proteomic analyses were performed to analyze the transcriptome and proteome changes at three crucial developmental stages: pre-cortex splitting, cortex splitting, and expansion, providing a valuable foundation for understanding the molecular basis of taproot development in radish (Mitsui et al., 2015; Yu et al., 2015, 2016; Xie et al., 2018). However, the molecular mechanisms underlying taproot thickening in radish remain partially clarified.

Phytohormones are crucial in the taproot thickening of radishes (Xie et al., 2018). GAs are essential classic phytohormones involved in various plant growth and development processes, including shoot elongation, root development, and xylogenesis (Castro-Camba et al., 2022). The effects of GA_3_ on tuber/root crops, such as potato (Xu et al., 1998), yam (Gong et al., 2015), sweet potato (Dong et al., 2019), carrot (Wang et al., 2015), and turnip (Liu et al., 2021), have been extensively studied recently, and GAs affect storage root formation and development differently. Several studies have indicated that exogenous application of GAs can inhibit the formation of storage organs that develop from the roots, stems, or stolons (Xu et al., 1998; Wang et al., 2015; Dong et al., 2019; Liu et al., 2021). Other studies have shown that the endogenous GA3 content is higher in the early expansion stage of yams, while exogenous GA_3_ increases the number of tubers, promotes their expansion, and increases their weight and yield (Gong et al., 2015, 2016). However, the distinct roles of GA_3_ in the root thickening stage in radishes remain unelucidated. In this study, changes in the morphology, cytology, hormone content, and gene expression of taproots treated with GA_3_ and PBZ were analyzed. Our study showed that exogenous GA_3_ significantly inhibited root thickening in a dose-dependent manner (**Figure 1**). The root fresh weight increased significantly after treatment with PBZ, while there was no significant difference in the root diameter, indicating that PBZ promoted root development in radish by increasing the root length rather than the root diameter, as evidenced by the taproot shape of PBZ-treated plants (**Figure 1**). Longitudinal and radical growth of taproots is precisely regulated by complex interactions among hormones in radishes. Therefore, we hypothesized that PBZ functions as an inhibitor of GA biosynthesis, disturbing the hormone balance and changing the shape of radish taproots.

The mechanisms by which GAs negatively regulate storage root formation differ in root/tuber crops. For example, GA inhibits potato development by preventing the transition from stolon to tuber (Xu et al., 1998), whereas the mechanism by which GA inhibits the formation of storage organs is similar among carrots, sweet potatoes, and turnips, involving the enhancement of xylem lignification (Wang et al., 2015; Singh et al., 2019; Liu et al., 2021). Our results showed that GA inhibits radish taproot thickening by inhibiting the formation and maintenance of the vascular cambium according to morphological and anatomical analyses (**Figure 2**), which is consistent with the results stating that cambial activity is strongly correlated with radial root growth and biomass in radishes (Jang et al., 2015).

### 3.2 GA_3_ inhibited taproot thickening by increasing the expression of IAA biosynthesis and signal transduction-related genes

The initiation and development of storage roots are synergistically regulated by endogenous phytohormones (Grandellis et al., 2016; Dong et al., 2019; Kolachevskaya et al., 2019). Consistent with this, transcriptome analysis and functional annotation revealed that many genes involved in the hormone signal transduction pathway were differentially expressed in response to GA_3_ and PBZ treatments, suggesting that exogenous GA_3_ signaling may interact with some node genes in the hormone signal pathway and regulate the expression of hormone signal-related genes, consequently inhibiting root thickening in radishes.

This study revealed that after GA_3_ treatment, the endogenous GA_3_ content and the expression of most GA_3_ downstream genes were increased, while GA3ox expression levels, a key GA synthesis gene, were significantly decreased (**Figures 3**, **7**). This indicates that taproots respond to exogenous GA_3_ by rapidly reducing endogenous GA_3_ synthesis, promoting its metabolism and enhancing its signal output. Auxin is essential for GA-mediated root growth, and IAA promotes primary root elongation by modulating the cellular responses to GA_3_ in *Arabidopsis* (Fu and Harberd, 2003). Auxin contents decreased significantly after expansion initiation and remained low at the mid-expansion and maturity stages in *Pueraria* (Xiao et al., 2023) and potato (Dong et al., 2019), indicating that auxin is involved in the transition of tuberous roots from longitudinal to radical growth and that low IAA concentration favors tuberous root expansion. In this study, we found that IAA contents were significantly increased after treatment with GA_3_ (**Figure 3**). Moreover, most auxin-synthesis and downstream-signaling genes, including Aux/IAAs, SAURs, and CH3s, were strongly induced by exogenous GA_3_ treatment (**Figure 7**). Therefore, we hypothesized that GA_3_ inhibits taproot thickening in radishes, mediated by the significant increase in endogenous IAA content and IAA signal transduction, and the high IAA content inhibits taproot thickening at the cortex splitting stage. The interaction of these two signaling pathways provides new insights into the regulation of taproot thickening in radishes. Further studies are required to identify the key genes that act as signaling hubs that incorporate the auxin-gibberellin signaling pathways.

### 3.3 GA_3_ retarded taproot thickening in radish by inhibiting the establishment and maintenance of vascular cambium

The cells in the vascular cambium divide and expand continuously to differentiate into the secondary xylem and phloem, causing the expansion of radish taproots (Zaki et al., 2012). Cambial activity is strongly correlated with taproot thickening, also referred to as radial or secondary growth, and biomass in radishes (Jang et al., 2015). Plant hormones are vital internal regulators of the establishment and maintenance of the vascular cambium. Auxin and its downstream signaling pathway genes, including Aux/IAA and Auxin Response Factor (ARF), reportedly promote cambial activity and vascular patterning (Miyashima et al., 2013). A previous study showed that most transcripts encoding Aux/IAA genes responsible for cambium secondary differentiation during radish taproot thickening were downregulated, whereas those encoding ARF genes were upregulated at the cortex splitting and expanding stages (Yu et al., 2016), further proving that these genes are involved in cambium secondary differentiation during radish taproot thickening. Downstream of the TDIF-PXY module, WOX transcription factors, WOX4 and WOX14, function redundantly to promote cambial activity in *Arabidopsis* (Hirakawa et al., 2010; Etchells et al., 2013). Downstream of the auxin signaling pathway, the auxin-dependent transcription factor MP/ARF5 promotes cambium activity by directly binding to the WOX4 promoter to activate WOX4 transcription (Brackmann et al., 2018). In this study, WOX4 and ARF5 expressions were induced by exogenous GA_3_ (**Figure 8**; **Supplementary Table S4**). Similarly, MP/ARF5 promotes PHB expression, an HD-ZIP-III, which maintains the stem cell organizer in a non-dividing state and promotes stem cell activity in adjacent vascular cambium cells (Brackmann et al., 2018). In contrast, PHB expression was suppressed by GA_3_ and PBZ treatments in this study (**Figure 8**; **Supplementary Table S4**). A previous study showed that WOX14 might function upstream of GA biosynthesis in *Arabidopsis*. The loss of WOX14 function results in GA-deficient phenotypes that can be complemented with exogenous GA application, whereas WOX14 overexpression stimulates the expression of GA3ox anabolic genes and represses GA2ox catabolic genes, promoting the accumulation of bioactive GA (Denis et al., 2017). Our data showed that GA_3_ treatment significantly inhibits the expression of WOX14 (**Figure 8**; **Supplementary Table S4**), possibly caused by an increased GA_3_ content, which inhibits the activity of the vascular cambium.

In the EPFL4/6-ER/ERL1 pathway, ER and its paralog ERL1, expressed in phloem cells, bind to their ligands, EPFL and EPFL6, and regulate cambial activity via an unknown pathway (Uchida et al., 2012; Uchida and Tasaka, 2013; Wang et al., 2019). Furthermore, in this study, the expression of ERL1 was significantly reduced by GA_3_ (**Figure 8**; **Supplementary Table S4**), further indicating that GA_3_ retarded root thickening by inhibiting cambial activity. GSK3 proteins interact with PXY and repress the transcription factor BES1, causing the inhibition of xylem cell differentiation (Kondo et al., 2014). However, in our study, BES1 expression was induced with GA_3_ and PBZ treatments (**Figure 8**; **Supplementary Table S4**). Furthermore, GA_3_ strongly suppressed the expression of xylem cysteine peptidase 2 (XCP2), whereas PBZ had the opposite effect and promoted the expression of XCP2 (**Figure 8**; **Supplementary Table S4**), which can inhibit xylem cell differentiation (Avci et al., 2008), indicating that XCP2 proteins can respond to GA_3_ signals, regulating root thickening in radish. These results indicate that there may be a multilayered regulatory network between these genes that respond to exogenous GA_3_ to inhibit taproot thickening. We speculate that the inhibitory effect on taproot thickening in radish was primarily achieved by inhibiting WOX14, ER/ERL1, and XCP2 expression to prevent the maintenance and differentiation of the vascular cambium.

In this study, integrated morphological, anatomical, hormonal and transcriptomic analyses were performed to determine the effects of GA_3_ and PBZ on taproot thickening and illustrate the mechanism by which GA_3_ regulates taproot thickening in radish. We found that GA_3_ significantly hindered root thickening by inhibiting the establishment of the vascular cambium. In addition, 3,050 DEGs were identified among three comparison groups. KEGG pathway enrichment analysis revealed that these DEGs were primarily involved in processes of biosynthesis of secondary metabolites and metabolic pathways. Furthermore, GA_3_ significantly increased the endogenous IAA content and upregulated the expression of IAA synthesis and signal transduction-related genes, including Aux/IAAs, SAURs, and CH3s. Therefore, we hypothesize that GA_3_ inhibits taproot thickening in radishes, mediated by a significant increase in endogenous IAA content and the expression of IAA biosynthesis and downstream genes. WOX14, ER/ERL1, and XCP2 also regulate taproot thickening significantly by responding to exogenous GA_3_ to regulate the maintenance and differentiation of the vascular cambium and can be used as core genes for in-depth investigations of the GA-mediated regulatory pathway of taproot thickening in radishes. These findings provide insights to elucidate the regulatory mechanism of GA_3_ on taproot thickening and accelerate the process of genetic improvement of radish taproots.

## 4 Materials and methods

### 4.1 Plant materials, treatments, and sample collection

The seeds of radish advanced inbred line, RB-203, were sown at the experimental station of the Crops Research Institute, Jiangsu Academy of Agricultural Sciences, Nanjing, China. The root cortex split of radish was achieved approximately 21 days after sowing, after which taproot thickening growth rapidly expanded (Wang et al., 2013). Subsequently, the radish seedlings growing at 21 days after sowing were foliar sprayed with 500 mL of aqueous solution containing GA_3_ (0.3 and 0.6 mM) or PBZ (0.1 mM), with the surface fully coated with liquid. Plants treated with distilled water were used as controls. Treatments were performed every 2 days for 2 weeks, and each treatment had three biological replicates, and each biological replicate containing 15 seedlings.

After 2 days of hormonal treatment, the thickest parts of seedling roots were collected, quickly placed in liquid nitrogen, and stored at -80°C for total RNA extraction and RNA-seq analysis. After 2 weeks of hormonal treatment, the seedlings were harvested for phenotypic analysis, anatomical structure analysis, and endogenous GA_3_ and IAA content measurements.

### 4.2 Phenotypic characterization

After 2 weeks of hormonal treatment, the shoot and root tissues of plants were sampled for phenotypic analysis and three biological replicates were prepared for each sample. The root diameter was measured at the thickest part of the root using vernier calipers. The fresh weights of the roots and shoots were measured using an electronic balance. After the above physiological indices of plants were detected, radish roots were collected and fixed for subsequent anatomical structure analysis or placed in liquid nitrogen for endogenous hormone level measurement.

### 4.3 Anatomical structure analysis

To analyze the changes in taproot structure after GA_3_ and PBZ treatments for 2 weeks, samples (1.0×0.5×0.5 cm) with three replicates of each treatment were collected from the thickest parts of radish roots and fixed in 4% paraformaldehyde dissolved in PBS (pH7.4) to maintain a natural status. The fixed samples were dehydrated with gradient ethanol and embedded in paraffin, as described previously (Liu et al., 2023). Samples were sectioned at a thickness of 10 µm with an RM 2145 microtome (Leica) and stained with 70% aqueous safranin O followed by 95% fast green FCF in absolute alcohol. Stained sections were observed, and images were captured using an OLYMPUS BX60 light microscope (Olympus, Japan).

### 4.4 RNA isolation, full-length cDNA library construction, and sequencing

Total RNA was isolated from the thickest parts of radish roots using the Quick RNA Isolation Kit (Huayueyang Biotechnology Co., Ltd., Beijing, China) following the manufacturer’s instructions. The quality of each RNA sample was monitored on 1% agarose gel. RNA purity was determined using a NanoPhotometer^®^ spectrophotometer (IMPLEN, CA, USA). RNA concentration was measured using Qubit^®^ RNA Assay Kit in Qubit^®^ 2.0 Fluorometer (Life Technologies, CA, USA), and the RNA integrity was checked using the Agilent Bioanalyzer 2100 system (Agilent Technologies, CA, USA). For each sample, an equal amount (1 μg) of total RNA was reverse transcribed into cDNA and used for cDNA library construction. The cDNA libraries were sequenced using an Illumina sequencing platform by Metware Biotechnology Co., Ltd. (Wuhan, China). After sequencing, clean reads were obtained by removing reads containing adaptors, paired reads with sequences having more than 10% unknown nucleotides, and reads with a quality rating of less than 50% (Q-value ≤ 20). The clean data analyzed in this study has been deposited in the National Center for Biotechnology Information (NCBI; project accession number: PRJNA1080271).

### 4.5 DEGs and enrichment analysis

The filtered clean reads were mapped to the reference genome of *R. sativus* (http://brassicadb.cn/#/SearchJBrowse/?Genome=Rapsa_Xiang_V1.0) using HISAT 2 (Kim et al., 2015). The expression level of each gene was measured using the fragments per kilobase exon model per million mapped reads (FPKM≥1) to normalize the calculation of gene expression. Differential expression analysis between sample groups was performed using DESeq2 to obtain DEG sets between two biological conditions (Love et al., 2014; Varet et al., 2016). After differential analysis, the false discovery rate was obtained by correcting the probability of hypothesis testing for multiple hypotheses using the Benjamini and Hochberg method (Benjamini and Hochberg, 1995). The screening criteria for DEGs were |log2Fold Change| ≥ 1.0 and false discovery rate < 0.05. To improve the DEG accuracy, genes with a fold change > 2 and Q-value ≤ 0.001 were defined as significant DEGs for DEGs identified in multiple comparisons. Furthermore, the DEGs were mapped to the Gene Ontology (http://www.geneontology.org) and KEGG databases to identify significantly enriched functional classifications and metabolic pathways. Significantly enriched gene ontology and KEGG terms were defined using Benjamini and Hochberg false discovery rate corrections at a P-value < 0.05.

### 4.6 Expression Analysis of DEGs Using RT-qPCR

RT-qPCR was performed to validate the RNA-seq data using 15 randomly selected genes. RNA was extracted as described above. The reverse transcription reaction (20 µl) was performed for the first cDNA strand synthesis using 1 µg of total RNA with a StepOnePlus™ Real-Time PCR System (Vazyme Biotech Co., Ltd., Nanjing, China). After completion of the reverse transcription reaction, the cDNA template was diluted 4-fold by adding 60 µl of ddH_2_O, and 1 µl of cDNA template was used for the RT-qPCR in a total volume of 20 µl. RT-qPCR was performed using the Fast SYBR Green Master Mix (Vazyme Biotech Co., Ltd., Nanjing, China) according to the manufacturer’s instructions. Three biological replicates were used for each experiment. Relative expression levels were calculated using the 2^-ΔΔCT^ method (Bustin et al., 2009). Specific primers for 15 genes were designed using Primer Premier 5.0 (**Supplementary Table S5**), and *RsActin2/7* and *RsTEF2* were used as the internal reference gene for the analysis of the 15 genes expression (Xu et al., 2012; Jang et al., 2015).

### 4.7 Determination of endogenous hormone contents

Cambial zone and inner parenchyma region of roots were collected respectively for endogenous hormone contents examination. Cambial zones can be visually distinguished from neighboring tissues because of their high cell density. These regions were carefully thin sectioned and collected. The concentrations of endogenous hormones were determined using an HPLC-MS/MS system comprising an AB Qtrap6500 triple quadrupole mass spectrometer (https://sciex.com.cn/) and an Agilent 1290 HPLC system (https://www.agilent.com/). A radish root sample (1 g) was ground into powder using liquid nitrogen and transferred to a glass tube. Subsequently, 10 mL extraction buffer (isopropanol/HCl) was added, followed by shaking for 30 min at 4°C, the addition of 20 mL dichloromethane (CH_2_Cl_2_), and shaking for another 30 min at 4°C. After centrifugation at 13,000 *g* for 5 min at 4°C, the organic phase was transferred to the glass vials and dried with nitrogen under dark conditions. The residue was dissolved with 400 µL of methanol and centrifuged at 13,000 *g* for 10 min at 4°C. Next, the supernatant was collected and filtered through 0.22 µm filters. The hormone concentrations in the solution were analyzed using ESI-HPLC-MS/MS (Grosskinsky et al., 2014). The liquid chromatography conditions were as follows: the reverse-phase chromatography column was Waters ACQUITY UPLC BEH C18 (100×2.1 mm, 1.7 µm) and column temperature 40°C, and the injection volume was 2 µL. Mass spectrometry conditions were as follows: curtain gas (35 psi), ion spray voltage (4500 V), atomization pressure (60 psi), auxiliary gas pressure (60 psi), and atomization temperature (500°C). Calibration curves for hormone quantification are listed in **Supplementary Table S6**. The measurements were performed in triplicates.

### 4.8 Statistical analysis

Data were analyzed with one-way ANOVA and Duncan’s test using SPSS Statistics (version 20.0; SPSS Inc., Chicago, IL, USA). Values in figures marked with different lowercase letters are significantly different at the 0.05 probability levels.

## Data Availability

The datasets presented in this study can be found in online repositories. The names of the repository/repositories and accession number(s) can be found below: PRJNA1080271 (SRA).
