# Supplementary material for: Exogenous gibberellin suppressed taproot secondary thickening by inhibiting the formation and maintenance of vascular cambium in radish (Raphanus sativus L.)
Source: Front Plant Sci. 2024 Sep 12;15:1395999. doi: 10.3389/fpls.2024.1395999 (PMC11424454; doi:10.3389/fpls.2024.1395999)
Supplement: Supplementary file 1 [file Table1.docx]

Supplementary Material

Article Title

Ge Meng^1^, Mingli Yong^1^, Ziyue Zhang^1^, Yuqing Zhang^2^, Yahui Wang^2^, Aisheng Xiong^2*^, Xiaojun Su^1*^

*** Correspondence:** Corresponding Author:
[xiaojunsu@jaas.ac.cn](mailto:xiaojunsu@jaas.ac.cn); [xiongaisheng@njau.edu.cn](mailto:xiongaisheng@njau.edu.cn).

# Supplementary Figures and Tables

## Supplementary Figures


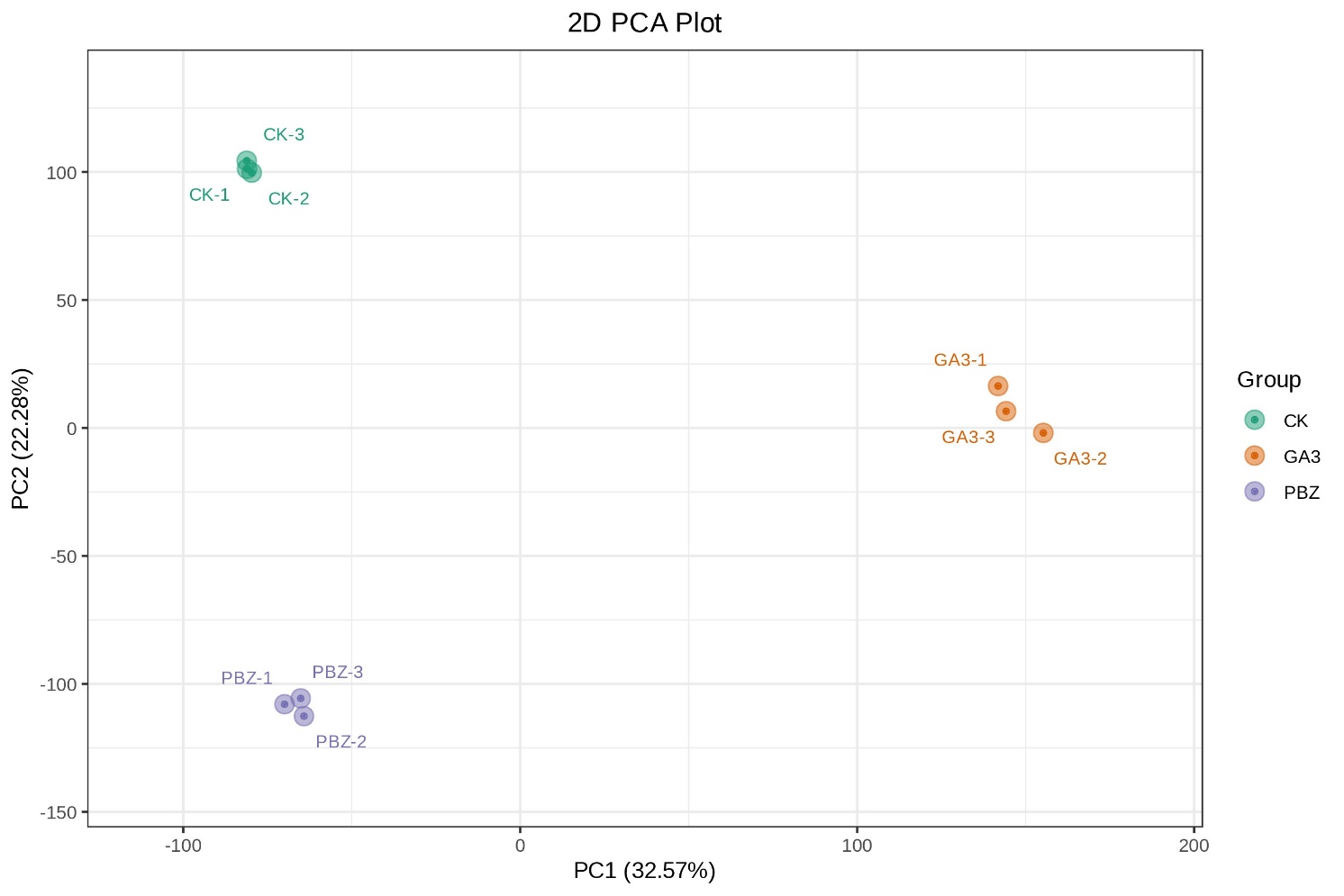


**Supplementary Figure 1.** PCA of RNA-seq data of different treatment.
